# Fact, Fiction, and Fitness

**DOI:** 10.3390/e22050514

**Published:** 2020-04-30

**Authors:** Chetan Prakash, Chris Fields, Donald D. Hoffman, Robert Prentner, Manish Singh

**Affiliations:** 1 Department of Mathematics, California State University, San Bernardino, CA 92407, USA; cprakash@csusb.edu; 2 Independent Researcher, 23 rue des Lavandières, 11160 Caunes Minervois, France; fieldsres@gmail.com; 3 Department of Cognitive Sciences, University of California, Irvine, CA 92697, USA; ddhoff@uci.edu (D.D.H.); robert.prentner@uci.edu (R.P.); 4 Department of Psychology and Center for Cognitive Science, Rutgers University, New Brunswick/Piscataway Campus, NJ 08854, USA

**Keywords:** natural selection, perception, veridicality, evolutionary psychology, Bayesian decision theory, fitness, evolutionary game theory, interface theory of perception

## Abstract

A theory of consciousness, whatever else it may do, must address the structure of experience. Our perceptual experiences are richly structured. Simply seeing a red apple, swaying between green leaves on a stout tree, involves symmetries, geometries, orders, topologies, and algebras of events. Are these structures also present in the world, fully independent of their observation? Perceptual theorists of many persuasions—from computational to radical embodied—say yes: perception veridically presents to observers structures that exist in an observer-independent world; and it does so because natural selection shapes perceptual systems to be increasingly veridical. Here we study four structures: total orders, permutation groups, cyclic groups, and measurable spaces. We ask whether the payoff functions that drive evolution by natural selection are homomorphisms of these structures. We prove, in each case, that generically the answer is no: as the number of world states and payoff values go to infinity, the probability that a payoff function is a homomorphism goes to zero. We conclude that natural selection almost surely shapes perceptions of these structures to be non-veridical. This is consistent with the interface theory of perception, which claims that natural selection shapes perceptual systems not to provide veridical perceptions, but to serve as species-specific interfaces that guide adaptive behavior. Our results present a constraint for any theory of consciousness which assumes that structure in perceptual experience is shaped by natural selection.

## 1. Introduction

If the experienced world of a neonate is unstructured, a “great blooming, buzzing confusion”, that of the adult is assuredly not. Consciously experienced visual space, for instance, has a non-Euclidean geometry [1,2,3,4]. Formal analyses of color experiences yields a variety of structures, including the RGB cube, the Schrödinger color solid, manifolds, fiber bundles, and the CIE xy-chromaticity space [5,6]. Visually experienced objects and surfaces admit description by differential geometry [7,8,9]. Experiences of sound intensity are ordered from soft to loud; pitch is ordered from low to high.

This is, of course, no surprise. The structure of experience has been the subject of experiments at least since the groundbreaking work, in the 1830s, of the physiologist Ernst Heinrich Weber. These investigations coalesced into a scientific field with the publication in 1860 of *Elements of Psychophysics,* by the physicist and philosopher Gustav Theodor Fechner. The goal of psychophysics is to investigate the structures of experience and their relationship to structures of the physical world. As Duncan Luce and Carol Krumhansl explain [10]:

[Psychophysics] anticipates the discovery of general laws relating the sensations to physical attributes. That is, the measured sensations are expected to correspond systematically to the physical quantities that give rise to them.

The systematic correspondences inferred from psychophysics experiments, which can be represented formally as maps from the observed/measured world to conscious experiences of the perceiver, are assumed to be homomorphisms. A homomorphism is a mapping that faithfully transports a structure, *I*, of a physical attribute of the world into a structure, Ψ(I), of the sensations. For instance, the physical attribute, *I*, of acoustic air pressure amplitude has the structure of a total order, from low amplitude to high amplitude. The corresponding sensation of auditory loudness, Ψ(I), is also a total order, from low to high. The relation between them, within the typical frequency range of the human auditory system, is a power law: $\Psi \left(I\right)=kI^{\alpha (f)}$ (here α(f)=0.67 for a 3000 Hertz tone, and *k* depends on the units used). This map transports, or respects, the total order: if acoustic amplitude $i_{1}$ is greater than $i_{2}$, then perceived loudness $\Psi (i_{1})$ is greater than $\Psi (i_{2})$, as long as one is within the human acoustic frequency range.

This form of power law, called Stevens’ power law in honor of the psychophysicist Stanley Stevens, holds for a variety of sensations, including vibrations, brightness, lightness, visual length, visual area, warmth, pain, tactile roughness and hardness, heaviness, and electric shock (for a critique, see [11,12]). The exponent in the power law depends, of course, on the sensation. But in each case the map from physical features of the world to sensations is also, within the typical dynamic range of the relevant human detection system, a homomorphism.

This is no minor point. Psychophysics assumes the existence of an observer-independent world, and moreover, one whose structure and function are those described by physics, even if no living creature perceives it. This assumption is tellingly illustrated by Einstein’s famous question to Abraham Pais about quantum theory. Pais recalls that he asked whether “I really believe(d) that the moon exists only when I look at it” [13]. If a sense is to inform us truly about the structure of such an observer-independent world (OIW), then the map from the OIW to experiences generated by that sense must not scramble or erase this structure. Only to the extent that the map approximates a homomorphism, can the sense inform us about the structure of the OIW. If a sense succeeds to inform us about the structure of the OIW, the resulting perceptions are called “veridical. ” Veridicality, in this usage, is “truth” in the traditional sense of a correspondence theory of truth in which a sentence is true if it reports the actual state of the OIW [14]. This assumption of veridicality is standard in the perceptual and cognitive sciences. This is made clear, among other things, by the fact that visual perception is standardly treated as implementing “inverse optics ”—namely, as a process that computes the most probable 3D world structure responsible for generating any given 2D retinal image(s) [15,16].

Psychophysics assumes that “physical variables,” including light intensity, acoustic amplitude, etc., are objective components of the OIW, and therefore, that descriptions of the OIW that are based on direct measurements of such physical variables provide a “ground truth” against which less-direct measurements can be validated. In this case, empirical evidence for psychophysical mappings (such as Stevens’ power law) from physical variables to experienced magnitudes or other outcomes is evidence that such experiences are homomorphic to structurers within the OIW, and hence are veridical. However, one can also argue that what we call physical variables are themselves the results of measurement procedures that observers conduct using their own perceptual and conceptual systems, and moreover, that the outcomes of such measurements are expressed either in terms of predicates that our perceptual representations employ, or simple generalizations of such predicates [17,18]. If this is the case, psychophysical mappings simply indicate systematic correspondences between two different forms of “measurements” that observers make—direct “physical” measurements (often implemented with specific measurement apparatus) and less-direct “sensory/perceptual” measurements. Such correspondences are non-trivial and interesting; however, they constitute homomorphic mappings between *different perceptual experiences*, not homomorphic mappings between perceptual experiences and the OIW. We return to this point when replying to objections in §5.

Even with the understanding that veridicality requires homomorphic mappings between the OIW and our experiences, most theories of perception (in both the sciences and philosophy of mind) conclude that some of our perceptions succeed in being veridical, and that we have natural selection to thank for this. They agree on this point, even though they disagree on other fundamental issues, such as whether perception involves representation or computation.

The neurophysiologist David Marr, for instance, argued that ”usually our perceptual processing does run correctly (it delivers a true description of what is there)” [19]. He ascribed this success to natural selection, claiming that we:

Definitely do compute explicit properties of the real visible surfaces out there, and one interesting aspect of the evolution of visual systems is the gradual movement toward the difficult task of representing progressively more objective aspects of the visual world.

Here “objective” means independent of any observer or observation: an “objective aspect of the visual world,” is a structure or state of the OIW. The philosopher Jerry Fodor was adamant that [20]:

There is nothing in the ’evolutionary,’ or the ’biological’ or the ’scientific’ worldview that shows, or even suggests, that the proper function of cognition is other than the fixation of true beliefs.

Fodor is here using “true” in the sense of correspondence referred to above. The cognitive scientist Zygmunt Pizlo concurs that [21]:

Veridicality is an essential characteristic of perception and cognition. It is absolutely essential. Perception and cognition without veridicality would be like physics without the conservation laws.

Each of these theorists proposes that perceptual systems process information, and that veridicality is achieved, in part, through sophisticated computations. Proponents of embodied cognition reject this proposal, and claim instead that natural selection achieves veridicality by shaping the joint dynamics of organism and environment. The philosopher and cognitive scientist, Anthony Chemero, for instance, says [22]:

OK, so (radical) embodied cognitive scientists can be realists. That is, they can believe that there is an animal-independent world, and that some of our perceptions and thoughts get it right.

Similarly, the philosopher Alva Noë and psychologist Kevin O’Regan conclude that “Perceivers are right to take themselves to have access to environmental detail” [23].

In what follows, we closely examine the claim that the structure of conscious experience is, at least some of the time, homomorphic to the structure of the presumed OIW, and hence can be regarded as, at least some of the time, veridical in the strong sense required by a correspondence theory of truth. We consider by far the most common argument for veridicality: that natural selection over evolutionary time will drive the perceptual systems of organisms to at least an approximation of veridicality. We formulate this argument in terms of evolutionary game theory and prove, under generic assumptions, that the probability that fitness payoff functions are homomorphisms of certain structures in the world approaches zero as the number of possible world states and potential payoff values become large. The structures we consider here are those of total orders, permutation groups, cyclic groups, and measurable spaces. These structures are critical for perceiving magnitudes (e.g., loudness, hardness, or heat as discussed above), rearrangements of objects, rotations and translations of objects, and probability distributions, respectively. As both Euclidean and well-behaved non-Euclidean geometries, including the experienced geometry of visual space, all respect rotational and translational invariants, structures implemented by cyclic groups are, in particular, crucial for the veridical perception of geometric space. The four theorems that we prove concern the information about these structures made available to perceivers by fitness payoff functions. These theorems are independent of any specific assumptions about perceivers or their structures, and are in particular independent of the organizational level or scale (e.g., as defined by [24,25]) at which selection acts.

These results show that unless novel, strongly restrictive (and non-circular) assumptions regarding the structures of fitness payoff functions are introduced, appeals to natural selection fail to support claims of veridical experiences. It is well understood that perception has limited range. The light that humans see, for instance, is only from a narrow band of the electromagnetic spectrum. What our theorems show is that our perceptions are not veridical even within the limited ranges where they do operate; i.e., they never faithfully report the structures in the observer-independent world even within those limits. The results are consistent with the interface theory of perception (ITP [17,18,26]), according to which natural selection shapes perceptual systems to evolve a species-specific interface to guide adaptive behavior, and not to provide veridical experiences of an objective reality. As such, the results present a constraint for any theory of consciousness which assumes that structure in perceptual experience is shaped by natural selection.

## 2. Natural Selection

The case for veridical perception is, as noted above, often based on natural selection. The core idea is that those of our predecessors who perceived the OIW more accurately had a competitive advantage over those who perceived it less accurately, and thus were more likely to become our ancestors, passing on their genes that coded for more accurate sensory systems. We are the offspring of such ancestors, so we have reason to be confident that our perceptions are, in the normal case, veridical.

The psychologist Stephen Palmer makes this case succinctly: “Evolutionarily speaking, visual perception is useful only if it is reasonably accurate” [27]. Evolutionary theorist Robert Trivers argues [28]:

Our sensory systems are organized to give us a detailed and accurate view of reality, exactly as we would expect if truth about the outside world helps us to navigate it more effectively.

Similarly, the psychologist Roger Shepard proposes that evolution shaped our senses to internalize various regularities of the external world. In his article “Perceptual-cognitive universals as reflections of the world” he claims [29]:

Natural selection has ensured that (under favorable viewing conditions) we generally perceive the transformation that an external object is actually undergoing in the external world, however simple or complex, rigid or nonrigid.

It is worth noting here that the assumption of an OIW underlies all of these statements.

However, some disagree, arguing that natural selection does not favor veridical perceptions. The philosopher Patricia Churchland claims instead that [30]:

Looked at from an evolutionary point of view, the principal function of nervous systems is [...] to get the body parts where they should be in order that the organism may survive [...] Truth, whatever that is, definitely takes the hindmost.

The cognitive scientist Steven Pinker agrees [31]:

Our minds evolved by natural selection to solve problems that were life-and-death matters to our ancestors, not to commune with correctness.

Later he concedes, however, that “we do have some reliable notions about the distribution of middle-sized objects around us” [32]. It is now widely understood that the primary selective forces in human evolution, at any rate, are social [33]. The “world” to which human perceptions are adapted is, therefore, not just the presumed OIW, but is also a world of other experiencing organisms. While the social character of the human world is often explicitly acknowledged (e.g., by Trivers [28]), the OIW is still regarded as the “ground truth” by theorists of veridical perception.

We have here a standoff. Natural selection is used by theorists to argue both for and against veridical perceptions. So which argument is correct? Assuming that natural selection governs the evolution of perceptual systems, we need not speculate whether it favors veridical perceptions or not. We can prove theorems. In the next section we review basic ideas needed to understand these theorems and their remarkable implications.

## 3. Evolutionary Games

Darwinian theory can be cast into a precise formulation in the mathematics of evolutionary game theory [34]. To understand evolutionary games, it helps to think of a video game in which a player grabs points. The reward is reaching the next level of the game. A variety of strategies are available to the player, including a choice of tools and tactics.

Players in evolutionary games can compete by employing different strategies to grab fitness payoffs; indeed, the most interesting games are games in which distinct strategies are deployed with equivalent skill. A strategy which collects, on average, more payoffs than its competitors is said to be fitter. The reward is reproduction—a new generation in which more players wield that strategy. While “evolution” is viewed as an optimization method in genetic algorithm based search [35,36], biological evolution is only satisficing [37,38]. This is reflected in evolutionary game theory by the assumption of an arbitrary payoff function, as opposed to a goodness-of-fit function with an a priori target [34,39].

But fitness payoffs depend heavily on context. Consider the fitness payoffs offered by eucalyptus leaves. For a hungry koala wanting to eat, they offer nutrition. For a sated koala wishing to mate, they offer nothing. For a hungry person wanting to eat, they offer death by cyanide. For a sated person wishing to mate, they offer nothing. The same leaves offer wildly different payoffs, depending on the organism (koala versus person), its state (hungry versus sated), and the action (eating versus mating). The key insight is that fitness payoffs depend on the combined state of the OIW—in this example the leaves—and the perceiver(s) inhabiting it: in this case the animals, their states, and their actions.

The domain of a “global fitness function” would therefore not be just the observer-independent world *W*, but the Cartesian product W×O×S×A, where *O* is the set of organisms, *S* their possible states, and *A* their possible action classes. Once we fix a particular organism o∈O, state s∈S, and action class a∈A, we then have a specific fitness function $f_{o,s,a}$ defined on *W* [17,18,40].

We can thus effectively represent the resulting (specific) fitness payoff function by a function that maps the states of the OIW, w∈W, into payoff values, v∈V. That is, for a fixed organism and action class, and suppressing the parameters o,s and *a*, we have a function:

(1) f:W→V.

Such payoff functions drive evolution by natural selection. They shape perceptions and actions. They determine whether natural selection favors veridical perceptions.

**Figure 1:**
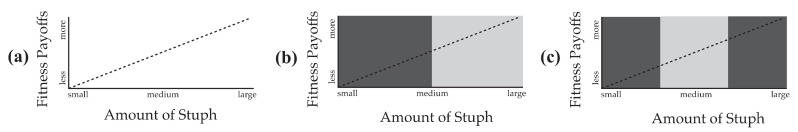
Assignments of fitness payoffs: (**a**) Fitness payoff is a linear function of the amount of stuph. (**b**) “Veridical” sensory map that is homomorphic to this function. (**c**) “Non-veridical” sensory map that is not homorphic to this function. It is less fit than the sensory map shown in (**b**).

We illustrate this with a simple example. The example also serves to highlight the important point that there need be no correlation between fitness payoffs and veridicality with respect to world (OIW) structure. Recall that the relevant notion of veridicality here (and the one standardly assumed in the perceptual and cognitive sciences) is indeed veridicality with respect to world (OIW) structure (see Section 1). Suppose the world has a resource, call it stuph, and a creature, call it kritre, that eats stuph. Kritres see just two colors, light gray and dark gray. As kritres forage for stuph, they choose where to eat by the colors they see. Suppose the payoff function assigns greater values to more stuph, as in Figure 1a. Consider a kritre that sees light gray if there is lots of stuph, and dark gray otherwise, as in Figure 1b. Its sensory map is a homomorphism of a total order – brighter color corresponds to more stuph. Thus its perceptions are veridical: they preserve the order structure of stuph in the world. This kritre has a simple way to reap greater payoffs: feed where it sees light gray. Consider a different kritre that sees light gray if there is a medium amount of stuph, and dark gray otherwise, as in Figure 1c. Its sensory map is not a morphism of a total order—darker color corresponds to more stuph and less stuph. Its perceptions are not veridical: they scramble the order structure of stuph in the world. This kritre has no way to consistently reap greater payoffs. If it feeds where it sees dark gray, it sometimes gets lots of stuph and other times gets little. It is less fit than the veridical kritre.

**Figure 2:**
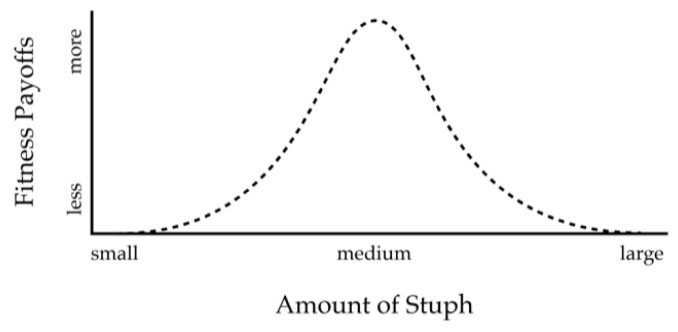
Payoff function that is a non-linear function of the amount of stuph. Now, the non-veridical sensory map of Figure 1c would be fitter than then sensory map of Figure 1b.

Suppose instead that the payoff function assigns greater values to medium amounts of stuph, as in Figure 2. Now the veridical kritre is in trouble. It has no way to consistently reap greater payoffs. If it feeds where it sees light gray, it sometimes gets a big payoff and other times gets a poor payoff. It has the same problem if it feeds where it sees dark gray. However the non-veridical kritre has a simple way to reap big payoffs: feed where it sees light gray.

What made the difference? The key is whether the payoff function itself is a homomorphism of the structure in the world. If it is, as in Figure 1a, then veridical perceptions are fitter, and natural selection favors them. If it is not, as in Figure 2, then veridical perceptions are not fitter. Instead, non-veridical perceptions that are homomorphisms of the payoff function are fitter, and natural selection favors them [41,42]. Thus, whether or not a fitness function is a homomorphism determines whether it can support veridical experiences—those that preserve structure in the observer-independent world *W*.

What about payoff functions that are not homomorphisms of structure in the world? Can they really occur? Or are they just abstract and implausible possibilities? As it happens, they occur often. Consider oxygen. Too little or too much is fatal to us. Only a narrow range of partial pressures of oxygen, between 19.5% and 23.5%, can sustain life. Thus the payoff function here is not a homomorphism: both low levels and high levels of oxygen map to low fitness values, whereas intermediate levels map to high fitness values. The same is true of ultraviolet radiation, blood glucose levels, and a host of other examples. This is no surprise. Life is delicate, requiring strict maintenance of homeostasis. So the corresponding payoff functions will not be homomorphisms of total orders.

Payoff functions can fail to be homomorphisms. But is this likely? If so, then selection is likely to favor non-veridicality; if not, then selection is likely to favor veridicality. If we can determine the probability that payoff functions are homomorphisms, then we can determine the probability that our perceptions are veridical.

That is the focus of this paper. We compute the probability that payoff functions are homomorphisms for four kinds of structures: total orders, permutation groups, cyclic groups, and measurable spaces. This tells us the probability that we perceive these structures veridically. The answer in each case is the same: the probability of veridical perception is zero.

We compute these probabilities by counting. We count all possible payoff functions, and count all payoff functions that are homomorphisms. We divide the number of homomorphisms by the total number of possible payoff functions to get the probability. (To be more conservative, we actually count only the total number of “admissible” fitness functions—those that achieve maximal fitness for at least some w∈W—and thus can truly shape selection processes; see next section.) These counts depend, of course, on the number of states of the world and the number of possible payoff values. But we find, for each structure, that in the limit as the number of world states and payoff values goes to infinity, the probability of homomorphisms goes to zero.

## 4. Four Theorems

We now present four theorems, one for each of four structures: total orders, permutation groups, cyclic groups, and measurable spaces. These structures correspond to perceptions of magnitudes, such as sound intensity or heat, re-arrangements of objects, rotations or spatial translations of objects, and probability distributions, respectively. Each theorem says the probability is zero that payoff functions are homomorphisms of the structure. Thus the probability of veridical perception of each structure is zero. We emphasize that these theorems concern the mathematical properties of the fitness payoff functions alone. They make no assumptions about, and are completely independent of, the cognitive architecture of the perceiving organism, including whether this architecture implements representations of any kind.

In each case, we compute the probability assuming that there are *n* states of the world and *m* possible payoff values. We then let *n* and *m* go to infinity to obtain our result.

Counting payoff functions that are homomorphisms is a bit tricky; we leave it for the proofs in the Appendix (for their definitions see Appendix A.1). But counting the total number of payoff functions is straightforward and is used in all four theorems. So we address it here.

The total number of payoff functions from a set of $N_{W}=n$ world states into a set of $N_{V}=m$ payoff values is simply $m^{n}$. The reason is that each world state can map to any one of *m* values, and there are *n* world states. So there are *m* possible values for the first world state, times *m* possible values for the second world state, …, times *m* possible values for the last world state. This is *m* multiplied by itself *n* times, i.e., $m^{n}$.

One might object, however, that this count includes payoff functions that are implausible, such as payoff functions in which every world state is assigned the lowest possible payoff value. How could natural selection occur with such a defective payoff function? Every strategy would be punished no matter what it did.

We think this objection is well taken. So we restrict our count to those payoff functions that take the maximum possible payoff value for at least one state of the world. For such payoff functions there are strategies that can reap maximum payoffs for at least one state of the world. We call these the admissible payoff functions.

To count the admissible payoff functions, we take the total number of payoff functions, which we computed above, and subtract the number of payoff functions that are not admissible. A payoff function is not admissible if it does not take the maximum payoff value. That means it only takes at most m−1 possible payoff values. The number of functions from *n* states of the world into a set of m−1 possible values can be computed by the same logic we saw two paragraphs ago. The number is ${\left(m-1\right)}^{n}$. Subtracting this from the total number of payoff functions, we find that the number of admissible payoff functions is $m^{n}-{\left(m-1\right)}^{n}$.

We now state our four theorems. The first theorem concerns total orders. It says, roughly, that most payoff functions are not homomorphisms of total orders, and thus that natural selection does not generically support veridical perception of total orders. More precisely, it says that as the number of world states and the number of payoff values increases, the probability goes to zero that admissible payoff functions are homomorphisms of total orders.

**Total Orders Theorem**. The number of admissible payoff functions that are homomorphisms of total orders is 2n+m−2m−1. Thus for any fixed *m*, the ratio between admissible homomorphisms of total orders and admissible payoff functions goes to zero as *n* goes to infinity. Additionally, even if we let *m* increase at the same rate as *n*, e.g., m=n, the ratio still goes to zero.

**Proof.** See Appendix A.2.

The second theorem concerns permutation groups, which preserve symmetry. Symmetry is ubiquitous in our perceptions, from the radial symmetry of an apple, to the bilateral symmetry of many animals, leaves, and human artifacts, to the roughly Euclidean symmetry of visual space. It seems natural to assume that these symmetries of perception faithfully present symmetries of the world, to assume [43]:

3D symmetrical shapes of objects allow us not only to perceive the shapes, themselves, veridically, but also to perceive the sizes, positions, orientations and distances among the objects veridically.

Our intuitive notion of symmetry is captured by the algebraic notion of a group [44]. A group is a set, *G*, together with a binary operation, ∘:G×G→G, that is associative ($\left(g_{1}\circ g_{2}\right)\circ g_{3}=g_{1}\circ \left(g_{2}\circ g_{3}\right),\;\forall g_{1},g_{2},g_{3}\in G$), has an identity element (∃e∈G,suchthatg∘e=e∘g=g,∀g∈G), and is such that each element has an inverse ($\forall g\in G,\exists g^{-1}\in G$, such that $g\circ g^{-1}=g^{-1}\circ g=e$). Some examples of groups are the real numbers under addition or, if 0 is excluded, under multiplication, the group $S_{n}$ of permutations of *n* objects, and the “general linear group” GL(n), the set of all n×n matrices under the operation of matrix multiplication. Other examples are subgroups of GL(n), such as the orthogonal matrices O(n) or the orthogonal matrices with unit determinant SO(n). In physics, important examples of subgroups of GL(n), where we allow the matrices to have complex entries, are the unitary matrices U(n), and the unitary matrices with unit determinant SU(n).

Here we investigate whether payoff functions are homomorphisms of symmetric groups. The symmetric group over any set is the group whose elements are all the bijections from the set to itself, and whose group operation is composition of functions. In the case of a finite set of *n* symbols, the symmetric group, $S_{n}$, consists of all n! possible permutations of the symbols.

Our second theorem says, roughly, that most payoff functions are not homomorphisms of symmetric groups, and thus that we do not have veridical perception of symmetry. More precisely, it says that as the number of states of the world and the number of payoff values increases, the probability goes to zero that payoff functions are homomorphisms of a symmetric group.

In the statement of the theorem, the number of world states is identical to the number of payoff values; i.e., n=m. If, as is usual, the number of world states exceeds the number of payoff values, we can think of the theorem as applying to a subset of, or a partition into, n=m world states that enjoy some symmetry.

**Permutation Groups Theorem**. The number of payoff functions that are morphisms of the symmetric group, $S_{n}$, is 2n+n! Thus the ratio of these to all admissible payoff functions is $\frac{2n+n!}{n^{n}-{\left(n-1\right)}^{n}}$, which has limit 0 as n→∞.

**Proof**. See Appendix A.3.

Our third theorem continues the study of symmetry. We look at cyclic groups, which are groups that can be generated by a single element. One example is the set, Z, of integers under addition; in fact, every infinite cyclic group is homomorphic to Z. Another collection of examples are the additive groups Z/nZ, the integers modulo *n*; every finite cyclic group of order *n* is homomorphic to Z/nZ. Cyclic groups appear, for instance, in the rotational symmetries of a polygon and the *n*-th roots of unity (roots of the polynomial $x^{n}-1$). The group of rotations of the circle $S^{1}$ is not cyclic; there is no rotation whose integer powers generate all rotations.

Our third theorem says, roughly, that most payoff functions are not homomorphisms of cyclic groups, and thus that we do not have veridical perception of cyclic symmetry. More precisely, it says that as the number of states of the world and the number of payoff values increases, the probability goes to zero that payoff functions are homomorphisms of a cyclic group.

**Cyclic Groups Theorem**. The number of payoff functions that are homomorphisms of the cyclic group is (m,n), the greatest common divisor of *m* and *n* [45]. The ratio of the number of cyclically homomorphic functions to admissible functions goes to zero as *n* goes to infinity and m≤n.

**Proof.** See Appendix A.4.

The fourth theorem concerns measurable spaces, which provide a framework for describing probabilities. Consider, for instance, flipping two coins. There are four possible outcomes, which we can write X={HH,HT,TH,TT}. If the coins are fair, each outcome has probability 1/4. We might also be interested in complex events, which are subsets of *X*. For instance, the event “at least one head” is the subset {HH,HT,TH}. If the coins are fair, this event has probability 3/4. A measurable space simply specifies a set of possible outcomes, *X*, and a set, X, of possible subsets of *X* called events, which includes all of *X* and is required to be closed under union and complement, i.e., to be an algebra; when *X* is countable, it is called a σ-algebra. Thus a measurable space is a pair (X,X). If *X* is finite, the largest algebra of events, X, is the set of all subsets of *X*, which is called the power set of *X* and sometimes denoted by $2^{X}$. It is called a discrete algebra. The smallest algebra of events consists of *X* and the empty set, and is called a trivial algebra.

In the case of measurable structures, the morphisms of interest are “reverse homomorphisms.” That is, if the world has a measurable structure (W,W) and payoff values have a measurable structure (V,V), then we are interested in functions f:W→V for which $f^{-1}$ is a homomorphism, mapping elements of V to elements of W. Such functions are called measurable. Measurable functions are of interest because they allow probabilities of events in the range to be informative about probabilities of events in the domain.

If W is discrete or trivial, or if V is trivial, then all functions f:W→V are measurable. However, in all other instances (i.e., those more relevant to perception), our fourth theorem says, roughly, that most payoff functions are not measurable, and thus that the probabilities of events in our experiences are not informative about probabilities of events in the world. More precisely, it says that as the number of world states and the number of payoff values increase, the probability goes to zero that payoff functions are measurable with respect to a large class of measurable structures. Each measurable structure in this class is characterized by the order *k* of its algebra, which is the minimal number of events which generate the entire algebra via disjoint union. For instance, if *W* has cardinality *n* and *W* is discrete, then k=n. But if *W* is generated by n/2 events, each event containing two outcomes, then k=n/2.

**Measurable Structures Theorem**. Suppose the measurable structure on *W* has order *k* and is neither trivial nor discrete. Additionally, suppose that the measurable structure on *V* is not trivial. Then the number of measurable functions is bounded by $m^{k-1}+{\left(\frac{m}{m-1}\right)}^{k-1}{\left(m-1\right)}^{n}.$

For most values of *k* the ratio of measurable payoff functions to all admissible payoff functions has limit 0 as n→∞.

**Proof**. See Appendix A.5.

## 5. Discussion: Does Natural Selection Favor Veridical Perceptions?

This is a technical question that can be addressed precisely using the theory of evolutionary games. Here we analyzed the payoff functions of evolutionary games, and showed that generically they are not homomorphisms of total orders, symmetric groups, cyclic groups, and measurable structures in the world. We conclude that if payoff functions erase these structures, then perceptions and actions shaped by these payoff functions cannot veridically present or represent these structures. Our proofs make no assumptions about the role of representations or computations in perception and action, so their conclusions apply equally to any computational, embodied, radical embodied, or Bayesian theory of perception and action that simply assume that senses evolve by natural selection. We wish to discuss a list of potential objections to our approach:

- We use the counting measure to prove that the probabilities of homomorphisms are zero. One might argue that this is the wrong measure. The main reason for using counting measure is that it is the canonical unbiased measure on finite sets of payoff functions. Proposing any specific biased measure would need careful explanation of why the logic of natural selection dictates this specific biased measure. We believe, however, that this burden cannot be met.
- The conclusions of our proofs are immune to the objection, “You cannot say whether something is veridical or not without first knowing what it is saying.” This objection assumes a representational account of perception, which is not required by our proof. Moreover, this objection is false on its face: an error-correcting code detects that a message received is not a veridical copy of the message sent, without knowing what the message is saying.
- One might wonder whether the theory of evolution can be an impartial arbiter in the debate over whether natural selection entails veridical perceptions. After all, does the theory itself not simply assume the veridicality of certain perceptions, such as organisms, species, physical resources, and (using some laboratory assay) DNA? How could the theory conclude against veridicality without refuting itself? This quandary has a simple solution, however. There is an algorithmic core to evolution by natural selection—variation, selection, and retention—which requires no commitment to DNA, organisms, and other such claims about the structure of the world. This algorithm, popularized as “Universal Darwinism,” applies to the evolution of organisms, but it has been speculated that it even applies to the evolution of art, music, memes, language, and social institutions [46,47].
- Our argument is based on evolution by natural selection. One can object that evolution is affected by many other factors—including genetic drift, pleiotropy, linkage, and constraints from physics and biochemistry—and that natural selection plays a relatively minor role.
  However, the standard evolutionary argument for veridical perceptions is that accurate perceptions are fitter, which is an argument from natural selection. To our knowledge, there are no arguments for veridical perception based on genetic drift, pleiotropy, linkage, or constraints from physics and biochemistry. Such arguments seem unlikely. It is hard to imagine how neutral drift, for instance, could favor veridical perceptions.
- Our argument focuses on just four structures: total orders, symmetric groups, cyclic groups, and measurable structures. There are, of course, many other structures relevant to perception, such as topologies, metrics, and partial orders. These structures also need to be studied, to see whether they are preserved by payoff functions. Ideally, one can hope for a general theorem, perhaps using category theory, that specifies all structures that are not preserved and thus not veridically perceived.
- One might object that many payoff functions are close to being homomorphisms of the structures of the world in, say, the sense of a $L^{2}$ norm, and thus that natural selection will shape perceptions to be close to veridical, if not precisely veridical. We reply that they will also be close to being homomorphisms of countless other structures that are not in the world, and thus that natural selection will equally shape perceptions to be close to countless non-veridical structures. There is no argument here for natural selection favoring perceptions that are close to veridical rather than close to countless non-veridical possibilities.

Our theorems show that perceptions are not veridical presentations of structures in the world, but do they show instead that perceptions are veridical presentations of fitness payoffs? Not at all. Natural selection finds satisficing solutions to adaptive problems. It does *not need* to be veridical. This gets particularly transparent if one considers that fitness is defined only relative to competition, modeled as an (evolutionary) game. If a cheap heuristic reaps more payoffs than the competition, then it is fit enough. Perception is veridical neither regarding the world, nor regarding fitness payoffs.

Instead, perception is more like a user interface [7,18,26,42,48,49]. A desktop interface hides the complex circuitry of a computer. It shows simple icons that let the user control the circuitry despite complete ignorance of the circuitry. That is what evolution has done for us. Space-time is our four-dimensional desktop, and physical objects are icons. They are not veridical presentations of the world. They are an interface that hides the world and guides adaptive interaction with that hidden world.

There are well-known cases of perceptions that code for fitness payoffs. The symmetry of a face, for instance, codes for reproductive potential [50]. The interface theory of perception says that such coding is ubiquitous: space-time and physical objects are data compressing and error correcting codes for fitness payoffs. They are satisficing solutions to the problem of compressing fitness payoffs into an actionable format. Physical objects are not veridical presentations of the world, but data structures that we create with a glance and garbage-collect with a blink.

## 6. Conclusions

Our intuitions rebel. Physical objects have a strong grip on the imagination. It is hard for us to imagine that the sight, smell, and texture of a red onion, which feel so real, which feel as though they present reality as it is, are instead just a data structure that we create as needed to guide adaptive action.

Fortunately, we have clear cases of this with some synesthetes. Carol Steen, for instance, sees a complex, three-dimensional object, with a clear color, motion, and surface texture, for each sound that she hears. She creates the object while she hears the sound, and then destroys it when the sound ceases. Each time she hears the same sound she creates the same object. This allows her to sculpt the object by replaying the sound until the sculpture is finished. She reports [51]:

These brilliantly colored and kinetic visions…are immediate and vivid…I work using just one ’sense trigger,’ such as sound…listening to only one selection of music at a time, played over and over again until the painting or sculpture is finished. A work need not be completed in one day provided I listen exactly to the same music when I return to work.

Michael Watson felt a complex, three-dimensional object with his hands each time he tasted something. Mint felt like tall, smooth, cool, columns of glass. Angostura bitters felt like a basket of ivy; Karo syrup like a tray full of ball bearings. He explained [52]:

When I taste something with an intense flavor, the feeling sweeps down my arm into my fingertips. I feel it—its weight, its texture, whether it’s warm or cold, everything. I feel it like I’m actually grasping something. Of course, there’s nothing really there. But it’s not an illusion because I feel it.

Evolution is likely not done with the perceptual interface of Homo sapiens. It is still tinkering. Here we see the data structures of physical objects given novel use in hearing and taste. This application is clearly not veridical. Ball bearings are not a veridical presentation of Karo syrup; ivy is not a veridical presentation of angostura bitters. The physical objects that we normally see when we open our eyes are, no less than these synesthetic objects, non-veridical data structures. They are just satisficing solutions to the problem of compressing and presenting fitness information for action, planning, and reasoning. 

## Abbreviations

The following abbreviations are used in this manuscript:

OIW	Observer-independent physical world
ITP	Interface theory of perception

## Appendix A. Proofs

### Appendix A.1. Definitions

Let *W* consist of all possible world states *w* and let *V* consist of all possible payoff values *v*.

**Notation** **A1.**
*For any natural number n, set $\underline{n}:=\left\{1,2,\ldots ,n\right\}$.*

We will take both spaces to be finite, so for some natural numbers n,m, we can represent *W* as a copy of $\underline{n}$ and *V* as a copy of $\underline{m}$. We are interested in fitness functions f:W→V or, equivalently, functions $f:\underline{n}\to \underline{m}$. In the following we will choose either, as convenient.

**Definition** **A1.** (**Quasi-Definition**). *A (“first-order ”) homomorphism of the same kind of structure in V and W is a function f:W→V that preserves this structure.*

This can take two forms, depending on the structure. For example, group structure is preserved under a “forward” homomorphism: If W,V are groups, a homomorphism f:W→V preserves the group multiplication, so that $f\left(w\cdot w^{\prime }\right)=f\left(w\right)\cdot f\left(w^{\prime }\right)$, for all $w,w^{\prime }\in W$. Other examples are linear homomorphisms preserving vector space structure, monotonic functions preserving order and open mappings preserving openness of sets.

On the other hand, a continuous function between topological spaces is a “backward” homomorphism: If a subset B⊂V is open in *V*, then $f^{-1}\left(B\right)$ is open in *W*. Another example of backward homomorphism is that of measurable functions preserving measurable structure.

We will need to define how one set *G* can act on another set *X* and how it can do so repeatedly. Suppose, firstly, we have the structure, in the acting set *G*, of an associative binary operation 〈·,·〉:G×G→G. This means that, for any pair g,h∈G we have 〈g,h〉∈G, which we call the *product* of *g* and *h* (in that order); we can write this simply as gh. Associativity means that for any g,h,k∈G, we have g(hk)=(gh)k. Such a pair (G,〈·,·〉) is called a *semigroup*. If the product is clear from context, we will simply write *G* for the pair (G,〈·,·〉).

A *monoid* is a semigroup possessing an identity: i.e., ∃ι∈G such that g·ι=ι·g=g.

A *group* is a monoid with all inverses: $\forall g\in G,\exists g^{-1}\in G:g^{-1}\cdot g=g\cdot g^{-1}=\iota$.

Now suppose, moreover, that each element of *G* behaves as an operation on another set *X*, i.e., each element of *G* is a function, moving every element of *X* to some other element of *X*. If the pair 〈G,·〉 is a semigroup, this allows us to be able to repeatedly “multiply” within *G*. The fact that each element acts on *X* means that the multiplication within *G* allows repeated such actions on *X*. We can think of the action of *G* on *X* also as a binary operation G×X→X, written as g·x for any g∈G and x∈X. We would like the two binary operations to be consistent. This leads us to the precise definition of an action of *G* on *X*:

**Definition** **A2.**
*Let G be a semigroup. We will say that G acts (as a semigroup) on a set X if there is a binary operation G×X→X, written as g·x for g∈G and x∈X, such that for any g,h∈G, and for any x in ∈X,*

(A1) g·(h·x)=(gh)·x.

*If G is a monoid, then we say that G acts as a monoid if it acts as a semigroup and also the identity ι acts, on all x in X, as*

(A2) ι·x=x.

*Finally, if G is a group which acts as a monoid, then it automatically acts as a group: ∀x∈X,∀g∈G,*

(A3) $$g^{-1}\cdot \left(g\cdot x\right)=\iota \cdot x=x.$$

*A homomorphism ϕ from G to H, where G,H are both semigroups, both monoids or both groups, is just a (forward) first-order homomorphism from G to H: ϕ:G→H is a homomorphism if ϕ(gh)=ϕ(g)ϕ(h),∀g,h∈G (the product on the left being in G and that on right being in H).*

In the following, we will assume that any *G* acting on a space will be a semigroup, a monoid, or a group. In this context, we introduce another kind of “homomorphism ”; namely, a *”second-order” homomorphism*. Suppose *G* acts on each of two sets X,Y. A second-order homomorphism is a pair, consisting of a homomorphism ϕ of *G* to itself, together with a function f:X→Y; these are mutually consistent in the following sense:

**Definition** **A3.**
*If G acts on sets X and Y, a second-order G-homomorphism from X to Y is a pair 〈ϕ,f〉 consisting of a homomorphism ϕ:G→G and a function f:X→Y, such that ∀g∈G and ∀x∈X, $f\left(g\cdot x\right)=\phi \left(g\right)\dot{f}\left(x\right)$.*

If the context makes it clear, we will abbreviate this by saying that “*f* respects ϕ. ”

A homomorphism of a semigroup, monoid or group *G* to another group *H* is just a (forward) first-order homomorphism from *G* to *H*: ϕ is a homomorphism if ϕ(gh)=ϕ(g)ϕ(h),∀g,h∈G.

Pictorially, the following diagram commutes, for all g∈G:

Finally:

**Definition** **A4.**
*A function $f:\underline{n}\to \underline{m}$ is admissible if it achieves its maximum value: there is some $k\in \underline{n}$ with f(k)=m.*

So admissible functions achieve the highest possible fitness value for some world state. In what follows we will compare the number of admissible homomorphic functions (in one of the above senses) to the number of all admissible functions, in the limit as the size *n* of the world grows to infinity.

### Appendix A.2. Total Orders Theorem: Counting Functions that are Monotonic, i.e., First-Order Homomorphisms Preserving (or Reversing) Order

The total number of functions $f:\underline{n}\to \underline{m}$ is $m^{n}$, since there are *m* possible values for each of the *n* elements of the domain. To count the admissible functions, we must remove those functions that do not achieve the value *m* for any element of *n*. By the same argument, the number of such functions, from $\underline{n}$ to $\underline{m-1}$ is now ${\left(m-1\right)}^{n}$. Thus, the total number of admissible functions is $m^{n}-{\left(m-1\right)}^{n}$.

Now suppose $\underline{n}$ and $\underline{m}$ have their natural order. What is the number of monotonic functions (i.e., either order-preserving or order-reversing functions)? We have:

**Lemma** **A1.**
*The number of monotonic functions $f:\underline{n}\to \underline{m}$ is given by 2n+m−1m−1 and the number of admissible monotonic functions is given by*

(A4) 2n+m−2m−1

**Proof.** Make a list of the first *n* natural numbers and insert m−1 vertical bars, each either before the list, somewhere in it, or after the list. To each way of doing this is associated a unique monotonically non-decreasing function *f* as follows: Any numbers before the first bar are given the value 1, those between the first and second bar the value 2, those between the j−1-th and *j*-th bar the value *j*, and so on, until the numbers (if any) after the last (m−1)-th bar, all given the value *m*. Clearly every non-decreasing function arises in this way, so the identification of such lists with non-decreasing functions is bijective. The number of ways of selecting m−1 places, for the bars, out of the n+m−1 numbers and bars is n+m−1m−1. For non-increasing monotonic functions, the count is exactly the same: just interpret the meaning of the numbers between bars in the reverse direction from *m* to 1. An admissible function achieves the value *m*, which in our identification means that the last bar in its list has at least the last number *n* after it (for non-decreasing functions; in the other instance, *n* is the first number). Upon removing *n* from consideration, we see that the admissible non-decreasing functions are in 1:1 correspondence with the lists of m−1 bars and n−1 numbers. Thus we are counting the number of ways of selecting m−1 spots out of the n+m−2 places. □

**Theorem** **A1.**
*For any fixed m, the ratio between the numbers of admissible monotonic functions and all admissible functions goes to zero as n goes to infinity. If we let m increase as n, i.e., m=n, the ratio still goes to zero.*

**Proof.** For any k≤n, we have nk≤nkk!. Thus the ratio of (A4) to all admissible functions is

(A5) 2n+m−2m−1/mn−(m−1)n≤2(n+m−2)m−1(m−1)!mn−(m−1)n.

For *m* fixed, no matter how large, this is eventually less than

(A6) $$\frac{2{\left(2n\right)}^{m-1}}{\left(m-1\right)!(m^{n}-{\left(m-1\right)}^{n})}=\frac{2^{m}}{\left(m-1\right)!(1-{\left(1-\frac{1}{m}\right)}^{n})}\cdot \frac{n^{m-1}}{m^{n}}$$

As *n* goes to infinity and since m>1, the first ratio goes to $2^{m}/\left(m-1\right)!$ As for the second ratio, applying L’hospital’s rule m−1 times shows that it has the same limit as does $\frac{(m-1)!}{{\left(\log m\right)}^{m-1}\cdot m^{n}}$, so our ratio goes to zero. Now set m=n. Robbin’s version of Stirling’s approximation [53] says that, for all *n*,

(A7) $$\sqrt{2\pi }n^{n+\frac{1}{2}}e^{-n}e^{\frac{-1}{12n+1}}<n!<\sqrt{2\pi }n^{n+\frac{1}{2}}e^{-n}e^{\frac{-1}{12n}}$$

Applying this to the ratio of (A4) to the number of admissible functions, we get

(A8) 22n−2n−1(nn−(n−1)n)=(2n−2)!(n−1)!(n−1)!·2nn−(n−1)n

(A9) $$\begin{array}{ccc} & < & \frac{\sqrt{2\pi }{\left(2\left(n-1\right)\right)}^{2\left(n-1\right)+\frac{1}{2}}e^{-2(n-1)}e^{\frac{-1}{12(2(n-1))}}}{{\left(\sqrt{2\pi }{\left(n-1\right)}^{\left(n-1\right)+\frac{1}{2}}e^{-(n-1)}e^{\frac{-1}{12(n-1)+1}}\right)}^{2}}\cdot \frac{2}{n^{n}-{\left(n-1\right)}^{n}}, \end{array}$$

which, after a little algebra, is $\frac{\exp[(36n-37)/24(n-1)(12n-11)]}{2\sqrt{\pi }}\cdot \frac{{\left(\frac{4}{n}\right)}^{n}}{\sqrt{\left(n-1\right)}\left(1-{\left(1-\frac{1}{n}\right)}^{n}\right)}$. As n→∞, the first ratio goes to $1/(2\sqrt{\pi })$ and since $1-{\left(1-\frac{1}{n}\right)}^{n}\to 1-\frac{1}{e}$, we see that again the whole expression goes to zero. □

### Appendix A.3. Permutation Groups Theorem: Counting Functions Preserving Symmetry under the Symmetric Group S n

We will take n=m and so only consider functions $f:\underline{n}\to \underline{n}$. We count the number of second-order homomorphisms of the symmetric group, acting on $\underline{n}$. These consist of certain functions, together with homomorphisms of $S_{n}$ to itself. We first classify the homomorphisms into three classes: within each class we count the number of functions respecting such homomorphisms and then sum over the three classes to get the total number of second-order homomorphisms. Then we compare this number with the admissible functions.

If $\phi :S_{n}\to S_{n}$ is a homomorphism of $S_{n}$, then by the first group isomorphism theorem, the image of ϕ is a subgroup of $S_{n}$, isomorphic to $S_{n}/\ker\phi$, where the *kernel* kerϕ of ϕ, is the set of elements sent to the identity by ϕ, and is a normal subgroup of $S_{n}$. Conversely, for any normal subgroup *K*, there is a canonical homomorphism from $S_{n}$ to $S_{n}/K$. So the set of homomorphisms of $S_{n}$ is in one-to-one correspondence with the set of all *automorphisms* of the groups $S_{n}/K$, as *K* ranges over the normal subgroups of $S_{n}$.

The normal subgroups of $S_{n}$ are, for n≥5, the trivial subgroup {ι}, the alternating group $A_{n}$ and the whole group $S_{n}$ (Corollary G.33 in [54], p.125). The corresponding quotient groups are isomorphic to $S_{n},Z_{2}$ and {ι} respectively. Finding all homomorphisms is then a matter of finding the automorphisms of each of $S_{n}$, $Z_{2}$ and {ι}.

The group of automorphisms of the quotient group $S_{n}$, for n≠6, is just the group of its *inner automorphisms* [55]; i.e., those of the form $h\mapsto g^{-1}hg$ for some fixed $g\in S_{n}$. Since different *g* yield distinct automorphisms, the size of this group is the same as that of $S_{n}$; i.e., n! (when n=6 the number of automorphisms is 2n!; Theorem 3.5 below remains true when *n* is at least 5). There is only one automorphism of the quotient group $Z_{2}$, namely, the identity automorphism, and the same is true of the quotient group {ι}.

We will denote the operation of an element *g* of the symmetric group $S_{n}$ on the element $k\in \underline{n}$ by g·k. Recalling Definition 1.4, a function *f* respectful of the homomorphism ϕ gives a second-order homomorphism 〈ϕ,f〉:

**Lemma** **A2.**
*There are n functions f that respect the trivial homomorphism.*

**Proof.** Since ϕ(g)=ι, we have that f(g·1)=f(1), for all $g\in S_{n}$. For any $k\in \underline{n}$ there is a *g* such that g·1=k, so *f* is constant. There are *n* constant functions. □

**Lemma** **A3.**
*There are n! functions $f:\underline{n}\to \underline{n}$ that respect some inner automorphism ϕ of $S_{n}$. Indeed, if ϕ is given by $\phi \left(g\right)=hgh^{-1}$, then f(k):=h·k is the only function respectful of ϕ.*

**Proof.** Given the automorphism $\phi \left(g\right)=hgh^{-1}$ and integer *j*, put f(j):=h·j. Then $f\left(g\cdot k\right)=h\cdot \left(g\cdot k\right)=\left(hg\right)\cdot k=\left(hgh^{-1}h\right)\cdot k=\left(hgh^{-1}\right)\cdot \left(h\cdot k\right)=\phi \left(g\right)\cdot f\left(k\right)$. Conversely, suppose $f:\underline{n}\to \underline{n}$ respects the inner automorphism $\phi \left(g\right)=hgh^{-1}$ for some fixed $h\in S_{n}$: i.e., $f\left(g\cdot k\right)=hgh^{-1}\cdot f\left(k\right)$. As *g* runs over the whole group, the right-hand side runs over the whole of $\underline{n}$, since the group action is transitive and $hgh^{-1}$ runs over the whole group. So *f* is onto, and being a function from a finite set to itself, is also 1:1. Thus *f* is itself a permutation, so of the form $f\left(k\right)=h^{\prime }\cdot k$ for some $h^{\prime }\in S_{n}$. Then, for any $g\in S_{n}$, $h^{\prime }\cdot g\cdot k=f\left(g\cdot k\right)=hgh^{-1}\cdot f\left(k\right)=hgh^{-1}h^{\prime }\cdot k$, or $h^{-1}h^{\prime }g\cdot k=gh^{-1}h^{\prime }\cdot k$. This being true for all $k,h^{-1}h^{\prime }g=gh^{-1}h^{\prime },\forall g$. So $h^{-1}h^{\prime }$ commutes with all *g*, which means that $h^{-1}h^{\prime }=e$. In particular, there is exactly one function respectful of any inner automorphism. Thus there are n! such functions. □

**Lemma** **A4.**
*The function $f:\underline{n}\to \underline{n}$ is respectful of a homomorphism onto an order-2 subgroup only if it is constant, so that there are n such functions.*

**Proof.** Suppose $H<S_{n}$ is of order two and $\psi :S_{n}\to H$ is a homomorphism. A function *f* respectful of ψ satisfies f(g·k)=ψ(g)·f(k). Since the kernel of ψ consists of $A_{n}$, the even permutations, we have that for $g\in A_{n},f\left(g\cdot k\right)=f\left(k\right)$: *f* is invariant under the action of $A_{n}$. Let $g=\left(1,k\right)\in A_{n}$. Then f(k)=f(1),k=1,⋯n: i.e., *f* is constant. □

Putting these facts together, we see that, for n≥5, the number of respectful functions is 2n+n!

**Theorem** **A2.**
*The ratio of respectful functions to admissible ones has limit 0 as n→∞.*

**Proof.** The ratio of respectful functions to admissible ones is $\left(2n+n!\right)/(n^{n}-{\left(n-1\right)}^{n})$ By Stirling’s approximation,

(A10) $$\begin{array}{ccc} \frac{2n+n!}{n^{n}-{\left(n-1\right)}^{n}} & ≃ & \frac{2n+\sqrt{2\pi }n^{n+\frac{1}{2}}e^{-n}}{n^{n}-{\left(n-1\right)}^{n}}=\frac{2n+\sqrt{2\pi }n^{n+\frac{1}{2}}e^{-n}}{n^{n}\left(1-{\left(1-\frac{1}{n}\right)}^{n}\right)} \end{array}$$

(A11) $$\begin{array}{ccc} & = & \frac{1}{1-{\left(1-\frac{1}{n}\right)}^{n}}\left(\frac{2}{n^{n-1}}+\frac{\sqrt{2\pi }n^{\frac{1}{2}}}{e^{-n}}\right). \end{array}$$

Since the first factor goes to $1/(1-e^{-1})$, the expression goes to zero as n→∞. □

### Appendix A.4. Cyclic Groups Theorem: Counting Functions Preserving Cyclicity on a Finite Group; or Periodic Functions on a Lattice

**Definition** **A5.**
*G is a cyclic group if there is a positive integer p such that $G=\{e,a,a^{2},\ldots ,a^{p-1}\}$, with $a^{p}=e$, the identity. p is the order of the group.*

Any such group is isomorphic to the additive group $Z_{p}$ under addition modulo *p*. The number of homomorphisms from $Z_{n}$ to $Z_{m}$ has been computed by Diaz-Vargas and Vargas de los Santos to be (m,n), the greatest common divisor of *m* and *n* [45]. Thus we have immediately:

**Theorem** **A3.**
*The ratio of the number of cyclically homomorphic functions to admissible functions goes to zero as n goes to infinity and m≤n.*

**Proof.** Since (m,n)≤n, we have, $\left(n,m\right)/(m^{n}-{\left(m-1\right)}^{n})\to 0$ as n→∞. □

### Appendix A.5. Measurable Structure Theorem: Counting Measurable Functions, that is, (Backward) Homomorphisms Preserving Algebra or Partition Structure

For finite sets, probabilities can be consistently defined on subsets called *events*, if the collection of such subsets forms an algebra. (Because our sets are finite, we do not need to deal with sigma-algebras in order to define probabilities. The results of this section do not hold for uncountable sigma-algebras. For example, the interval (0,1) on the Borel real line is not a countable union of elementary sets, so Proposition 5.4 does not hold.)

**Definition** **A6.**
*An algebra W on the set W is a collection of subsets that includes the empty set and is closed under intersections and complements. (W)-measurable sets are the members of the algebra and the pair (W,W) is called a measurable space. Given an algebra V on another set V, a function f:W→V is W/V measurable if, for every V-measurable set T, $f^{-1}\left(T\right)$ is W-measurable.*

As a consequence of the definition, the whole set is measurable, as are unions and differences of measurable sets. Measurable functions are then (backward) homomorphisms of the measurable, or algebra, structure. In this section we will compare the number of measurable, admissible functions to the totality of admissible functions and find its limit as its size *W* goes to infinity.

In the following, we shall assume that (W,W) and (V,V) are finite measurable sets.

**Definition** **A7.**
*Given any $j\in \underline{n}$, let $U_{j}$ be the smallest measurable set containing j, i.e., the intersection of all such sets.*

Because of finiteness, $U_{j}\neq \emptyset$, for any *j*.

**Lemma** **A5.**
*If $j\in \underline{n}$ and $k\notin U_{j}$, then $U_{j}\cap U_{k}=\emptyset$.*

**Proof.** Were *j* to be also an element of $U_{k}$, $U_{k}\setminus U_{j}$ would be a measurable set containing *k* but not *j* which is strictly smaller than $U_{k}$, contradicting the latter’s minimality. So we have $j\notin U_{k}$. Suppose $U_{j}\cap U_{k}\neq \emptyset$. Since $j\notin U_{k},j\notin U_{j}\cap U_{k}$. Thus $j\in U_{j}\setminus U_{k}$, but this is a contradiction as, by hypothesis, $U_{j}\setminus U_{k}$ is a measurable set containing *j* yet strictly smaller than the minimal $U_{j}$. □

**Proposition** **A1.**
*Algebras on $\underline{n}$ are in 1:1 correspondence with partitions of $\underline{n}$, consisting of the minimal measurable sets of Definition 5.2. In particular, a general measurable set is a (disjoint) union of some of those in the partition.*

**Proof.** Let A be an algebra on $\underline{n}$, with minimal sets $U_{j}$ and let $j\in \underline{n}$. If any $U_{j}=\underline{n}$ we are done. If not, there is a $k\notin U_{j}$ and by the lemma, $U_{j}\cap U_{k}=\emptyset$. Continuing in this way, every $j\in \underline{n}$ is represented, so there is a finite subset $\left\{j_{1},\ldots ,j_{k}\right\}\subset \underline{n}$ such that ${\left\{U_{j_{i}}\right\}}_{i=1,\ldots k}$ is a partition of $\underline{n}$. Let *U* be a measurable set. If *U* is empty, it is the empty union of of the sets in this partition. If *U* is non-empty, pick any element of *U*, call it $j_{1}$. By minimality, $U_{j_{1}}\subset U,U\setminus U_{j_{i}}$ is measurable and is either empty or contains some element, say $j_{2}$. We have that $j_{2}\notin U_{j_{1}}$ so $U_{j_{2}}\cap U_{j_{1}}=\emptyset$. Continuing in this way through the finite set $\underline{n}$, we see that, for some m≤n, *U* is a union of the disjoint measurable sets in the partition: $U={⋃}_{i=1}^{m}U_{j_{i}}$. □

Note that if *W* is countable and ,W is a σ-algebra, the conclusion of this theorem holds, by induction.

**Definition** **A8.**

1. *The collection of subsets in the partition corresponding to the algebra W on W will be termed the base of the algebra and will be written as $\left\{W_{1},\ldots ,W_{k}\right\}$.*
2. *The order of an algebra is the number of sets constituting its base. For example, the order of the trivial algebra is 1, and the order of the discrete algebra is the size of the underlying set.*
3. *The characteristic of an algebra is the multiset giving the sizes of each of the elements $W_{i}$ of the base: we say that the characteristic is ${\left\{m_{i};l_{i}\right\}}_{i}$ if there are $m_{i}$ subsets of size $l_{i}$. Thus $\sum_{i}m_{i}l_{i}=n$, where n is the size of W. (In other words, the characteristic of an algebra is a partition, in the usual sense, of the number n. Saying that two algebras have the same characteristic is an equivalence relation on the collection of algebras: either algebra can be obtained from the other by a simple renumbering, or permutation, of the set W.)*

Let the base of the algebra W on *W* be the partition $\left\{W_{1},\ldots ,W_{k}\right\}$ and the base of the algebra V on *V* be the partition $\left\{V_{1},\ldots ,V_{l}\right\}$. Saying that f:W→V is W/V measurable is equivalent to saying, by Proposition 5.4, that for each base set $V_{j}\in V$, we have $f^{-1}\left(V_{j}\right)={⨆}_{i=1}^{p}W_{j_{i}}$, for some *p*-tuple of positive integers $\left(j_{1},\ldots ,j_{k}\right)$, where 1≤p≤k and ⨆ means “disjoint union.” So *f* is measurable if the inverse image of the partition of *V* (made up of the base of *V*) is a new, coarse-grained, partition of *W* (made up unions of elements of the base of W).

Note that when W is discrete (i.e., the minimal measurable sets are all the singletons), *all* functions f:W→V are W/V measurable and their total number is therefore $m^{n}$. And this is also true of all functions when V is trivial (i.e., the only measurable sets are *∅* and *V*): the number of W/V measurable functions f:W→V is again $m^{n}$. More generally:

**Lemma** **A6.**
*For any given algebra V, the number of W/V measurable functions f:W→V is determined solely by the order, and not the characteristic, of the algebra W. (The number of such measurable functions may, however, depend on the details of the algebra V, including its order m, as we will see below.)*

**Proof.** We have seen above that this is true when V is trivial, so suppose V is not trivial: i.e., its base consists of two or more subsets. If now W is discrete, there is only one characteristic of W (of order *n*), so the number $m^{n}$ is determined by the order *n*. Again, if W is trivial, there is only one characteristic of W (of order 1); a little thought shows that the number of measurable functions is now determined, and solely by the characteristic of V. So suppose in the following that W is neither discrete nor trivial. Thus let the base of W be $\left\{W_{1},\ldots ,W_{k}\right\}$ with n>k>1. Since W is not discrete, we can suppose that there is a base set, by renumbering call it $W_{1}$, of size at least two. This set has the form $W_{1}=\left\{a,b,\cdot \right\}$. Since W is not trivial, there is a distinct nonempty base set, call it $W_{2}$. We may suppose $W_{2}=\left\{c,\cdot \right\}$ (where, in either set, · represents zero or more elements!). We make a new algebra $W^{\prime }$, also of order *k*, by taking only the element *b* and moving it to $W_{2}$: the base of $W^{\prime }$ is $\left\{W_{1}^{\prime },\ldots ,W_{k}^{\prime }\right\}$ with $W_{1}^{\prime }=\left\{a,\cdot \right\},W_{2}^{\prime }=\left\{b,c,\cdot \right\}$ and $W_{i}^{\prime }=W_{i},\;i>2$. We will call such a move: *Basic Move*: {a,b,·},{c,·}…↦{a,·},{b,c,·}… Notice that the order of the algebra has not changed, but its characteristic certainly has. □

We will show that

**Claim 1**: Any algebra of order *k* can be obtained from any given one by means of a finite sequence of steps of the above kind.

**Proof.** Let us say that elements *a* and *b* are *companions* in the algebra A if they belong to the same base set of A. Suppose we have two algebras $A_{1}$ and $A_{2}$, both non-discrete, with the same order but different characteristics. We can convert one to the other using the following two-step algorithm. In the first step, suppose that {a} is a singleton in $A_{2}$, but not in $A_{1}$. Then we can use basic moves to remove all of its $A_{1}$-companions, to any other basic set there, to get the new algebra $A_{1}^{\prime }$, thus producing the singleton {a} as a new basic set. While repeating this procedure for subsequent singletons, remove their putative companions to basic sets other than the singletons already created. In this way, we can produce all the singleton sets that are basic in $A_{2}$. Of course, there may be extra singletons left over from $A_{1}$, but these will be dealt with in the next step. In the second step, whenever *a* and *b* are companions in the algebra $A_{2}$, but *not* companions in $A_{1}$, we can bring them together into a single (new) basic set by means of one of two processes:

1. If at least one of *a* and *b* belong to multi-element sets of $A_{1}$, we can perform a basic move, as above, to bring them into the same basic set of the new algebra $A_{1}^{\prime }$.
2. If both of *a* and *b* belong to singleton sets of $A_{1}$, then because of the non-discreteness, there is another base set *C* with 2 or more elements. Pick an element c∈C. Make these elements companions by performing three basic moves in sequence, as follows:
  {a},{b},C…↦{a},{b,c},C∖{c}…↦{a,b},{c},C∖{c}…
  Of course, C∖{c} is not empty, so these moves preserve the order of the new algebra.

Having brought together $A_{2}$-companions *a* and *b*, we continue to use basic moves to bring all their other $A_{2}$-companions into the same basic set. Once this is done, we move to a different basic set containing an element, at least one of whose $A_{2}$-companions is not in that set. Now iterate the procedure for that element and all of *its* companions not already in the same basic set. Each of the operations involved maintains the algebra order. Two distinct collections of $A_{2}$-companions could not end up being in the same basic set, because that would reduce the order by 1. When all operations are completed, we will have arrived at $A_{2}$. □

**Claim 2**: If W and $W^{\prime }$ have the same order, then the number of W/V measurable functions is the same as the number of $W^{\prime }/V$ measurable functions.

**Proof.** We will show that the number of W/V measurable functions that are not $W^{\prime }/V$ measurable equals the number of $W^{\prime }/V$ measurable functions that are not W/V measurable. This establishes that the number of measurable functions is the same for both algebras and the theorem will be proved once this claim is established. Assume that W and $W^{\prime }$ are the algebras related by a single basic move of the kind preceding claim 1. Recall that $b\in W_{1}$ and $c\in W_{2}$, where $W_{1}$ and $W_{2}$ are distinct base sets. For *f* a W/V measurable function, let $X_{1}$ and $X_{2}$ be the base sets of V such that $f\left(b\right)\in X_{1}$ and $f\left(c\right)\in X_{2}$. First, suppose $X_{1}$ and $X_{2}$ are the same set. Then $f^{-1}\left(X_{1}\right)=W_{1}⊔W_{2}⊔W^{0}$, where $W^{0}$ is union of base sets disjoint from both $W_{1}$ and $W_{2}$, so that $W^{0}\in W\cap W^{\prime }$. But $W_{1}⊔W_{2}=W_{1}^{\prime }⊔W_{2}^{\prime }$ so $f^{-1}\left(X_{1}\right)=W_{1}^{\prime }⊔W_{2}^{\prime }⊔W^{0}\in W^{\prime }$. All other $V_{i}$ in the base of V are disjoint from $X_{1}=X_{2}$, so the sets $f^{-1}\left(V_{i}\right)$ are unions (possibly empty) of base sets $W_{j}$ other than $W_{1}$ and $W_{2}$; these being both in *W* and $W^{\prime }$, *f* is also $W^{\prime }/V$ measurable. As we have seen in the proof of claim 1 this will establish claim 2 whenever W and $W^{\prime }$ are oft the same order. Next, assume $X_{1}$ and $X_{2}$ are not the same, and therefore disjoint. We will show that then $f^{-1}\left(X_{1}\right)\notin W^{\prime }$, so that *f* is not $W^{\prime }/V$ measurable. Now $f^{-1}\left(X_{1}\right)=W_{1}⊔W_{1}^{0}$, where $W_{1}^{0}$ is a union of base sets disjoint from both $W_{1}$ and $W_{2}$ and so $W_{1}^{0}\in W\cap W^{\prime }$. Similarly, $f^{-1}\left(X_{2}\right)=W_{2}⊔W_{2}^{0}$, where $W_{2}^{0}$ is disjoint from both $W_{1}$ and $W_{2}$ and so $W_{2}^{0}\in W\cap W^{\prime }$. Additionally, all other $f^{-1}\left(V_{i}\right)$ are unions (possibly empty) of base sets $W_{j}$ other than $W_{1}$ and $W_{2}$. Now $f^{-1}\left(X_{1}\right)=W_{1}⊔W_{1}^{0}=W_{1}^{\prime }⊔\left\{b\right\}⊔W_{1}^{0}$. But note that $W_{2}^{\prime }$ being a minimal set of $W^{\prime }$, $\left\{b\right\}⊊W_{2}^{\prime }$ is not in $W^{\prime }$. Assume $f^{-1}\left(X_{1}\right)=W_{1}^{\prime }⊔\left\{b\right\}⊔W_{1}^{0}$ is in $W^{\prime }$. Since $W_{1}^{\prime }⊔W_{1}^{0}\in W^{\prime }$ and $b\notin W_{1}^{\prime }⊔W_{1}^{0}$, we would then have $\left\{b\right\}=f^{-1}\left(X_{1}\right)-\left(W_{1}^{\prime }\dot{\cup }W_{1}^{0}\right)\in W^{\prime }$, a contradiction. So *f* is not W/V measurable. Put $v_{1}=f\left(a\right)\in X_{1},v_{2}=f\left(b\right)\in X_{1}$, and $v_{3}=f\left(c\right)\in X_{2}$. By disjointness, $v_{3}\neq v_{1},v_{3}\neq v_{2}$. Let $f^{\prime }$ satisfy $f^{\prime }\left(a\right)=v_{3},f^{\prime }\left(b\right)=v_{1},f^{\prime }\left(c\right)=v_{2}$ and $f^{\prime }\left(x\right)=f\left(x\right),\;x\notin \left\{a,b,c\right\}$. Then, by an argument, mutatis mutandis that above, $f^{\prime }$ is $W^{\prime }/V$ measurable but not W/V measurable. Thus the number of $W^{\prime }/V$ measurable functions is at least that of the W/V measurable ones. Reversing the roles, mutatis mutandis, of W and $W^{\prime }$, we see that the number is the same and the claim is proved. □

As the order of V increases by refinement, the number of measurable functions decreases:

**Lemma** **A7.**
*Fix the algebra W on W and assume it is not discrete. Let $V,V^{\prime }$ be algebras on V such that $V^{\prime }$ is a refinement of V: each base set of $V^{\prime }$ is contained in a base set of V. If the order of $V^{\prime }$ is strictly greater than that of V, then the number of $W/V^{\prime }$ measurable functions is strictly less than the number of W/V measurable functions.*

**Proof.** Let the order of V be l<m, with base $\left\{A_{1},\ldots ,A_{l}\right\}$. An algebra $V^{\prime }$ of order l+1 can be made from V by extracting a nonempty proper subset from a base set of V that has at least two elements, thus creating two new sets. By an appropriate renumbering, let us call the original set $A_{1}$ and the piece that is removed $A_{l+1}^{\prime }$. The two new sets thus created are $A_{1}^{\prime }$ := $A_{1}\setminus A_{l+1}^{\prime }$ and $A_{l+1}^{\prime }$. If we take $A_{i}^{\prime }=A_{i},\;i=2,\ldots ,l$, then the base of the new algebra $V^{\prime }$ is $\{A_{1}^{\prime },\ldots ,A_{l+1}\}.$ Now if *f* is a $W/V^{\prime }$ measurable function, it is automatically W/V measurable: for 2≤i≤l, $f^{-1}\left(A_{i}\right)=f^{-1}\left(A_{i}^{\prime }\right)\in W$, while, $f^{-1}\left(A_{1}\right)=f^{-1}\left(A_{1}^{\prime }⊔A_{l+1}^{\prime }\right)=f^{-1}\left(A_{1}^{\prime }\right)⊔f^{-1}\left(A_{l+1}^{\prime }\right)\in W$. Thus the number of $W/V^{\prime }$ measurable functions is no greater than the number of W/V measurable functions. Let $W_{0}$ be a base set of W which has two or more elements. Single out one element $x\in W_{0}$ and consider any function that takes all of $W_{0}\setminus \left\{x\right\}$ into $A_{1}^{\prime }$, takes *x* to $A_{l+1}^{\prime }$ and takes each remaining $W_{i}$ into $A_{1}^{\prime }$. Such a function is W/V measurable but not $W/V^{\prime }$ measurable, so the decrease in number is strict. □

Observe that the collection of algebras on *V* is a lattice, ordered by $V\geq V^{\prime }$ if $V^{\prime }$ is a refinement of V. The proof above shows that the number of measurable functions is monotonically increasing with this partial order. Across algebras on *V* with the same order *l*, the number of measurable functions could be widely different. But as *l* increases to *m*, the size of *V*, that number will decrease, in each maximal linearly ordered sublattice, to a lowest number: that for the discrete algebra on *V*.

We seek an upper bound on the number of measurable functions. By Lemma 5.7, we need to maximize, given any fixed characteristic of a non-discrete algebra on *W*, this number over all possible characteristics of algebras on *V* of smallest order, i.e., order two.

**Theorem** **A4.**
*Suppose the measurable structure on W has order k and is neither trivial nor discrete. Additionally, suppose that the measurable structure on V is not trivial. Then the number of measurable functions f:W→V is bounded by*

(A12) $$m^{k-1}+{\left(\frac{m}{m-1}\right)}^{k-1}{\left(m-1\right)}^{n}.$$

**Proof.** Let $W=\underline{n}=\left\{1,\ldots ,n\right\}$, and suppose W has base $\left\{W_{1},\ldots ,W_{k}\right\}$, for 2≤k≤n−1. Lemma 5.6 allows us to choose any characteristic of order *k*. Let us choose one with as many singletons as possible: $W_{1}=\left\{1,\ldots ,n-k+1\right\}$ consists of the first n−k+1 elements, and for 2≤j≤k, $W_{j}$ is just the singleton {n−k+j}. Suppose, for the highest possible count, that the base of V is $\left\{V_{1},V_{2}\right\}$, such that $|V_{1}|=m_{1},\left|V_{2}\right|=m_{2}=m-m_{1}$. For f:W→V to be measurable, we need, for some subset A⊂{1,…,k},

(A13) $$f^{-1}\left(V_{1}\right)=\underset{i\in A}{⋃}W_{i};\;\;f^{-1}\left(V_{2}\right)=\underset{i\in A^{\prime }}{⋃}W_{i},$$

where $A^{\prime }$ is the complement of *A*. Distinguish two instances:

(i) 1∈A. Then $f^{-1}\left(V_{1}\right)=W_{1}\cup C$ for C⊂{n−k+2,…,n} and $f^{-1}\left(V_{2}\right)=C^{\prime }$, where $C^{\prime }$ consists of the remaining elements of *W*: i.e., $C^{\prime }:=\left\{n-k+2,\ldots ,n\right\}\setminus C$.
(ii) 1∉A. Then $f^{-1}\left(V_{1}\right)=C^{\prime }$ for C⊂{n−k+2,…,n} and $f^{-1}\left(V_{2}\right)=W_{1}\cup C$.

In either instance, the number of such functions is a product of two counts, summed over all possible *C* of size *l*, l∈{0,…,k−1}. Once we have computed the count for instance (i), that for instance (ii) is that same count with $m_{1}$ and $m_{2}$ interchanged. Consider instance (i). First, the count for $f^{-1}\left(V_{1}\right)$: count the number of all functions from $W_{1}$ to $V_{1}$; times the number of functions from any fixed *C* of size *l*, l∈{0,…,k−1}, to $V_{1}$. This is

(A14) $$m_{1}^{n-k+1}m_{1}^{l}=m_{1}^{n-k+1+l}.$$

Second, the count for $f^{-1}\left(V_{2}\right)$ is the number of all functions from $C^{\prime }$ to $V_{2}$. This is

(A15) $$m_{2}^{k-1-l}.$$

Finally, we multiply by the number of subsets *C* of size *l* (of a set of size k−1) and sum. The total count for instance (i) is then

(A16) ∑l=0k−1k−1lm1n−k+l+1m2k−1−l.

Setting i=k−l−1, this is the same as

(A17) m1n∑i=0k−1k−1im2m1i.

For instance (ii), we interchange the roles of $m_{1}$ and $m_{2}$, so that the total number we seek is

(A18) m1n∑i=0k−1k−1im2m1i+m2n∑i=0k−1k−1im1m2i.

By the binomial theorem this sums to

(A19) $$m_{1}^{n}{\left(1+\frac{m_{2}}{m_{1}}\right)}^{k-1}+m_{2}^{n}{\left(1+\frac{m_{1}}{m_{2}}\right)}^{k-1}=m^{k-1}\left(m_{1}^{n-k+1}+{\left(m-m_{1}\right)}^{n-k+1}\right).$$

A little calculus shows that this has an extremum only at $m_{1}=m/2$, at which its value is a minimum. Thus the maximum occurs at the end-points $m_{1}\in \left\{1,m-1\right\}$, at both of which this count is

(A20) $$m^{k-1}+{\left(\frac{m}{m-1}\right)}^{k-1}{\left(m-1\right)}^{n}.$$

□

**Corollary** **A1.**
*For fixed m≥2, the ratio of the number of measurable functions to admissible functions goes to zero as n goes to infinity.*

**Proof.** Since $m^{k-1}/\left(m^{n}-{\left(m-1\right)}^{n}\right)\to 0$ and ${\left(m/\left(m-1\right)\right)}^{k-1}$ stays constant, we just need to check the limit of ${\left(m-1\right)}^{n}/\left(m^{n}-{\left(m-1\right)}^{n}\right)={\left({\left(m/\left(m-1\right)\right)}^{n}-1\right)}^{-1}$ which, for m≥2, goes to zero. □

**Remark** **A1.**
*For m large and fixed, this goes to zero slowly as n goes to infinity. If we allow m to grow fast enough, as O(n), say m=n, then the limit is 1/(e−1)≈0.58.*
